# Keep Calm: Does parental preoperative anxiety affect post-tonsillectomy pain scores in children?

**DOI:** 10.1007/s00405-024-08683-0

**Published:** 2024-05-13

**Authors:** Clara Serdoura Alves, Joana Dias, Sara Azevedo, Francisco Sousa, Mariline Santos, Joao Lino, Luis Meireles

**Affiliations:** Centro Hospitalar Universitário do Porto, Porto, Portugal

**Keywords:** Pediatric otolaryngology, Tonsillectomy, Postoperative recovery, Parental anxiety

## Abstract

**Purpose:**

To understand if high parental anxiety leads to increased post-tonsillectomy pain in children.

**Methods:**

Prospective study including parents of children aged 3–10 years old submitted to tonsillectomy with or without adenoidectomy. To evaluate anxiety, parents were asked to fill the State-Trait Anxiety Inventory form-Y, with postoperative pain being evaluated with the Wong-Baker FACES pain scale at postoperative days 1, 3 and 7. Parents were also asked to register the number of days during which children took analgesic and the number of analgesic intakes needed.

**Results:**

41 parents were enrolled, of which 95.1% (n = 39) were female with a mean age of 35.64 years (SD 5.751), with 41 children also being enrolled, of which 85.4% of children (n = 35) underwent tonsillectomy and adenoidectomy. 43.9% (n = 18) of parents presented State anxiety scores above the cut-off level and 53.7% (n = 22) above the Trait anxiety scores above the cut-off. Children of parents with high State anxiety presented statistically higher pain scores in both the third (p = 0.035) and the seventh postoperative days (p = 0.006), with significantly longer use of analgesic medication (p = 0.043) being found, as well as a statistically higher number of analgesic intakes (p = 0.045) (Table 4).

**Conclusion:**

The present study establishes an association between preoperative parental anxiety, postoperative pain scores and the need for longer analgesic use in children undergoing tonsillectomy. This reinforces the importance of reducing parental anxiety and opens the door for further strategies to better post-tonsillectomy outcomes.

## Introduction

Many children and their parents develop significant stress and anxiety in the preoperative period [1]. The relationship between preoperative anxiety and postoperative outcomes has been extensively studied in the adult population, with studies suggesting that high preoperative anxiety leads to a more difficult and prolonged postoperative recovery [1]. Scarcer information is available regarding this association in the pediatric population [2], with information about the relation between parental anxiety and postoperative outcomes in children being even more limited [3], with Rosenberg et al. reporting that higher parent preoperative anxiety is associated with higher infant postoperative pain scores and Sarkisova et al. reporting high parental anxiety as a predictor of narcotic use in adolescents following Spinal Fusion [3, 4].

Pediatric tonsillectomy, whether combined with adenoidectomy or not, is one of the most commonly performed surgical procedures in the world, being the most common surgery performed by otolaryngologists in the United Kingdom and following only Myringotomy with insertion of tubes in the United States [5, 6]. While commonly performed, Tonsillectomy is still associated with a myriad of complications, one of which is oropharyngeal pain, with prolonged sore throat potentially leading to decreased oral intake, dehydration, dysphagia or even hospital readmission [5].

Therefore, understanding what factors may contribute to increased and/or prolonged postoperative oropharyngeal pain after tonsillectomy is of the utmost importance, with this study aiming to understand if high parental anxiety leads to increased post-tonsillectomy pain in children.

## Methods

The present study was conducted at Centro Hospitalar Universitário de Santo António between March and September 2023 and was approved by the local ethical committee with patients’ rights being protected in compliance with the ethical principles of the Declaration of Helsinki and with all parents providing informed written consent.

Parents of children aged 3–10 years old scheduled to be submitted to elective tonsillectomy (with or without adenoidectomy) in an outpatient setting were asked to be part of the study. Exclusion criteria included refusal to partake in the study, not being fluent in the Portuguese Language, having underlaying psychiatric disorders, or having taken anxiolytic and/or anti-depressive medications in the 72h prior to taking our questionnaire.

To evaluate parental anxiety levels, the *State-Trait Anxiety Inventory form Y* (STAI form-Y) was given to parents before surgery. The STAI form-Y is a widely used instrument in clinical settings to diagnose anxiety, being made up of two sections: the first 20 questions evaluate State anxiety, or anxiety in a given moment, using items that measure feelings of apprehension, tension, worry and nervousness “right now”; and the second set of 20 questions assesses Trait anxiety, or baseline anxiety, including general sates of calmness, confidence and security. State anxiety questions are presented before the Trace anxiety set of questions, with all questions being scored from 1 to 4. Scores in both sets therefore range from 20 to 80 points, with higher values correlating with greater anxiety. This questionnaire has been previously validated for the Portuguese population by Silva and Spielberg [7] (Appendix 1), and high parental anxiety was defined as 1 Standard Deviation (SD) above the normative mean, with State anxiety considered for values equal and over 48 and Trait anxiety for values equal and over 47.

Pain in children submitted to surgery was evaluated using the Wong-Baker FACES pain rating scale. The Wong-Baker FACES pain scale was originally developed for the pediatric population and uses number ranges in the context of facial expressions as a visual representation of the patient’s pain experience [8].

Children and parents’ demographic data and information regarding previous anesthetic and surgical procedures in both children and parents was also collected.

Statistical analysis was performed using SPSS (IBM Corp., Armonk, NY), and p-values below 0.05 were considered statistically significant. A descriptive analysis of patients’ characteristics was performed considering frequencies (for categorical variables) and mean and standard deviation (SD) (for continuous variables). Normal distribution was assessed using the Shapiro-Wilks test and through analysis of the skewness and kurtosis. Differences among paired groups were evaluated with the use of chi-square test (for categorical variables) and independent sample t test or Mann–Whitney test (for continuous variables).

## Study protocol

### Recruitment

Parents were recruited on the day of the intervention, between one to two hours before the procedure, with all parents being asked about any impending doubts about the intervention and/or about postoperative care. After a brief explanation of both sets of questions, he STAI form-Y questionnaire was performed.

### Operation room (OR) and post-anesthesia care unit

All parents accompanied their children inside the OR and remained by their side until anesthetic induction, being accompanied by a nurse to another room afterwards. During the procedure, all children received a dexamethasone dose of 0.5 mg/kg, with a maximum dosage of 10 mg. All patients were monitored for any adverse effects in the Postanesthesia Care Unit.

### Before discharge

All patients were discharged on the day of the surgery. Before discharge, all parents received an analgesic prescription for their kid, comprised of paracetamol, prescribed to be taken every 6 h, at a dose of 15 mg/kg per take, only if pain was reported by the child. Parents were also given the Wong-Baker FACES pain rating scale, to be applied at home at postoperative days 1, 3 and 7, as well as an analgesic diary, spanning from postoperative day 1 to postoperative day 15, to document whether the prescribed analgesic was needed in each day and, if so, how often.

Children were postoperatively evaluated at day 15 with the Wong-Bakers FACES scale and the analgesic diary being collected at this time: pain scores were registered in the data base for day 1, 3 and 7; the number of times the prescribed dose of paracetamol was administered in each day was also registered, as well as the total number of days it was used.

## Results

### Study population

All parents of otherwise healthy children undergoing elective outpatient tonsillectomy (either alone or in association with adenoidectomy) and aged 3–10 years were included in this study. All interventions were done by the same surgeon, and thus only parents of children intervened by this doctor were asked to participate, with all surgeries being done by cold dissection and pilar suture. No parent presented any exclusion criteria, and thus 41 parents were enrolled, of which 95.1% (n = 39) were female with a mean age of 35.64 years (SD 5.751), with 31.7% of parents having completed the 12th grade and 31.7% having university studies (Table 1). 68.3% (n = 28) of parents had had a previous surgical and/or anesthetic intervention performed on themselves, with 1 parent (2.4%) experiencing complications from this intervention. 56.1% (n = 23) of parents had children who had previously undergone surgery, with no parent reporting complications of their children intervention.

**Table 1:** Parents study population

		Education	
Gender and age		4th grade	4.9% (n = 2)
Female (95.1%, n = 39)	Male (4.9%, n = 2)	9th grade	26.8% (n = 11)
35.64 (SD 5.751)	34.50 (SD = 3.536)	12th grade	31.7% (n = 13)
		College degree	31.7% (n = 13)
		Master’s degree	4.9% (n = 2)
		Total	100% (n = 41)

41 children were also enrolled, 21 of which from the male sex and 20 from the female sex, with a mean age of 5.19 (SD 1.940) and 4.85 (SD 1.694) respectively, with 78% of children undergoing surgery because of pediatric Obstructive Sleep Apnea. Of all interventions, 85.4% of children (n = 35) underwent tonsillectomy and adenoidectomy. No complications from the surgical interventions were reported and no children required emergency evaluation after tonsillectomy (Table 2).

**Table 2:** Children study population

	Mean age
Female	48.8% (n = 20); 4.85 (SD 1.694)
Male	51.2% (n = 21); 5.19 (SD 1.940)
Total	100% (n = 100)

### STAI form-Y

**Fig. 1:**
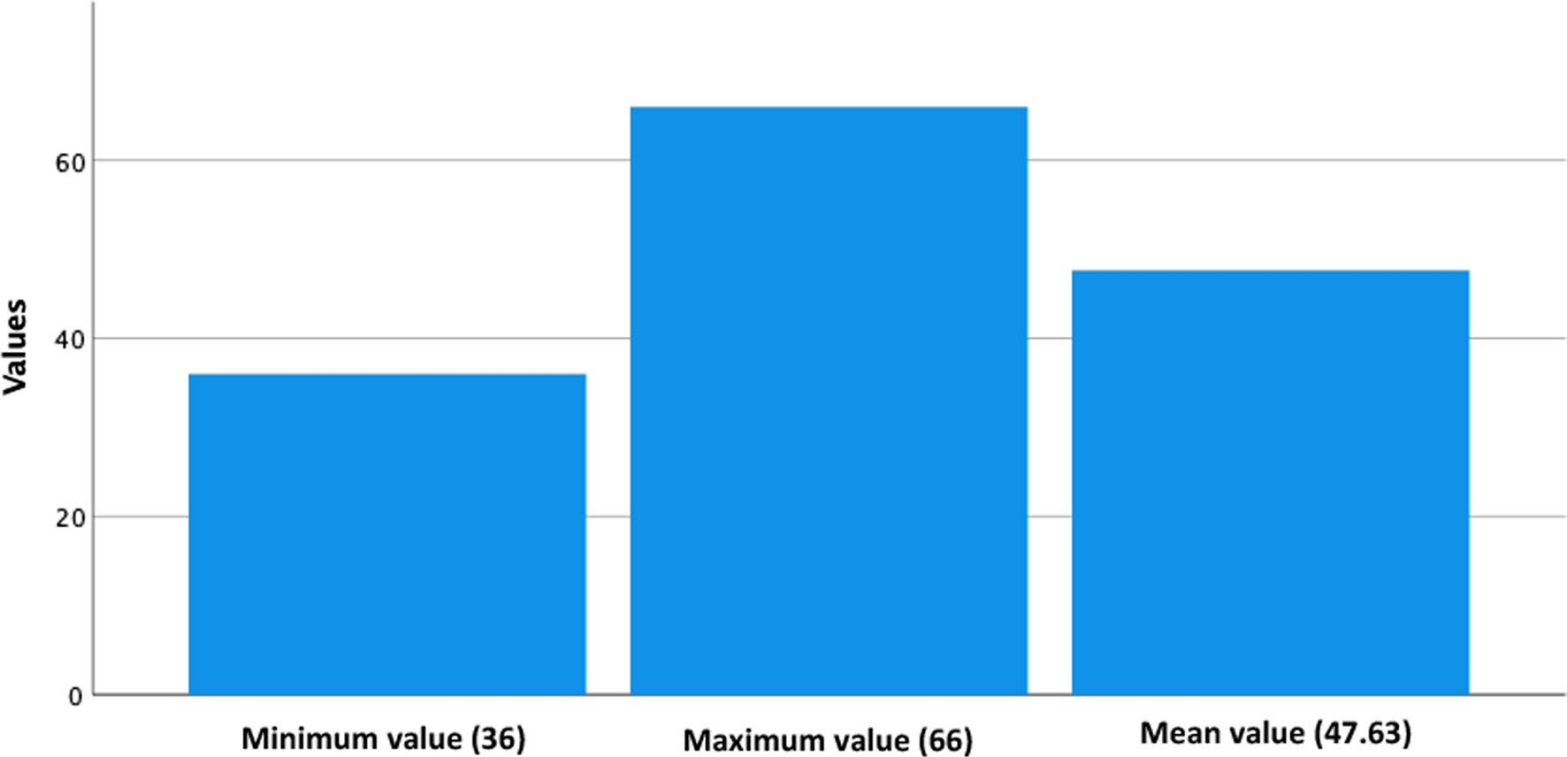
State anxiety minum, maximun and median scores

**Fig. 2:**
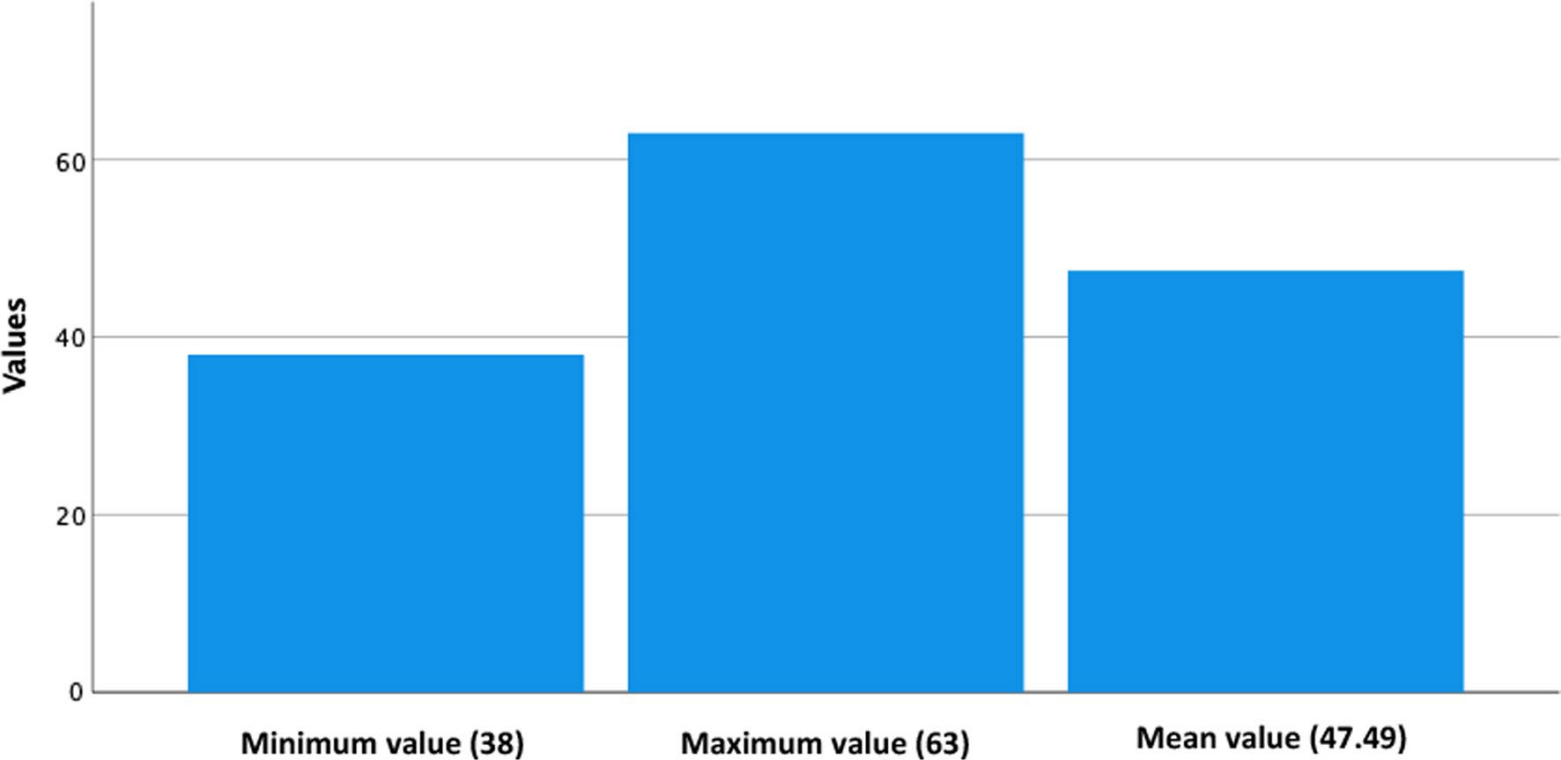
Trait anxiety minum, maximun and median scores

Parents presented a mean score of 47.63 in the State set of questions of the STAI form-Y and a mean score of 47.49 in the Trait set of questions, with scores ranging from 36 to 66 and from 38 to 63, respectively (Figs. 1 and 2). Overall, 43.9% (n = 18) of parents presented State anxiety scores above the cut-off level, with 53.7% (n = 22) presenting Trait anxiety scores above the cut-off.

No statistically significant differences were found between State or Trait anxiety scores between mother and fathers or in parents with a personal history of surgery or whose children had previously undergone a surgical procedure. No statistically significant difference was found between State or Trait anxiety scores between parents with higher education (college or master’s degree) and parents without higher education (Table 3).

**Table 3:** STAI state and trait mean scores

Parent surgery				Previous children surgery				Education			
State anxiety		Trait anxiety		State anxiety		Trait anxiety		State anxiety		Trait anxiety	
Surgery	No surgery	Surgery	No surgery	Surgery	No surgery	Surgery	No surgery	Higher education	Mandatory education	Higher education	Mandatory education
47.71	47.46	47.54	47.38	47.65	47.61	47.39	47.61	46.80	48.12	47.13	47.69
p = 0.906		p = 0.928		p = 0.984		p = 0.888		p = 0.522		p = 0.728	

### Parental anxiety, children pain scores, total days of analgesic use and total analgesic intakes

Postoperative pain scores presented mean values of 3.44 (SD 1.534), 2.10 (SD 1.546) and 0.68 (SD 1.035) for the first, third and seventh postoperative days, respectively. The prescribed analgesic was used for a mean of 4.24 (SD 3.231) days, with values ranging from 0 to 13 days post-surgery. The total number of analgesic administrations ranged from 0 to 22 (mean of 8.22, SD 6.162).

Children of parents with high State anxiety presented statistically higher pain scores in both the third (p = 0.035) and the seventh postoperative days (p = 0.006), with significantly longer use of analgesic medication (p = 0.043) being found, as well as a statistically higher number of analgesic intakes (p = 0.045) (Table 4).

**Table 4:** Pain scores, number of days and total analgesic intakes between parents with and without state and trait anxiety

	State anxiety		p value	Trait anxiety		p value
	Yes	No		Yes	No	
Day 1	3.72	3.22	0.301	3.36	3.53	0.740
Day 3	2.67	1.65	**0.035**	2.23	1.95	0.570
Day 7	1.17	0.3	**0.006**	0.86	0.47	0.234
Number of days taking analgesic	5.39	3.35	**0.043**	4.14	4.37	0.822
Total analgesic intakes	10.39	6.52	**0.045**	7.95	8.53	0.771

Bold numbers indicate statistical significance

No statistically significant differences were found between pain scores, total days of analgesic use or total number of analgesic administrations in children of parents with high Trait anxiety and of parents without high Trait anxiety.

No differences were found in children submitted to tonsillectomy alone or paired with adenoidectomy, between children’s sexes or between mothers and fathers.

## Discussion

The preoperative experience is a well-documented source of anxiety for both children and their parents [9]. While a common response, excessive preoperative anxiety may have a harmful impact on postoperative outcomes and on the clinical postoperative course, with preoperative anxiety in children shown to result in negative postoperative behavior, longer hospital stays and even long-term behavioral problems [10, 11].

While the relation between children preoperative anxiety and postoperative outcomes has been studied, information regarding the effect of parental anxiety on postoperative outcomes in children is limited [12].

The main goal of the present study was, therefore, to assess if parental anxiety affects post-operative outcomes, namely pain and analgesic use, in children undergoing tonsillectomy.

Our results show that children of parents with high State Anxiety present higher postoperative pain scores for both postoperative days 3 (2.63 vs 1.65, p = 0.035) and 7 (1.17 vs 0.3, p = 0.006). This is an indicator that not only does high parental State Anxiety lead to higher pain degrees but also to a longer postoperative recovery in children, something also supported by the longer analgesic intake by these children (5.39 vs 3.35, p = 0.043) and a higher number of analgesic intakes (10.39 vs 6.52, p = 0.045).

While these differences were found for parents with high State anxiety, higher Trait anxiety scores didn’t have an impact on children pain scores for any of the assessed days or on the number of days using analgesic or number of analgesic intakes. While State anxiety refers to a current state of anxiety, or the response to an event perceived as adverse on a given moment, Trait anxiety refers to more stable aspects of anxiety, including states of calmness, confidence and security [13, 14]. This reinforces the idea that it is, in fact, the anxiety felt by parents at the preoperative time that impacts postoperative pain and recovery in children, and not overall anxiety.

Tonsillectomy has been identified as one of the most painful surgical procedures, with pediatric tonsillectomy being associated with a painful and lengthy recovery period [15, 16]. Although many studies have been conducted on the management of post-operative pain, with a multimodal approach of paracetamol paired with an NSAID as the most recommended analgesic approach, pain control remains a clinical challenge [5, 15]. The findings of the present study indicate that approaching and managing parental preoperative anxiety might be an additional strategy in reducing postoperative pain, with studies showing that several actions have a positive impact on preoperative parental anxiety [9, 10]. Preoperative parental education, namely the use of informational leaflets and counseling, has been found to reduce parental anxiety significantly in children undergoing surgery for congenital heart disease and children undergoing ENT surgery [9]. To the same extent, therapies using play-based interventions, such as clown therapy, have been shown to reduce preoperative parental anxiety, as have interventions using music [9]. While these strategies have been shown to reduce parental preoperative anxiety, further studies are needed to understand it these results would successfully lead to reduced pain scores and reduced need of post-operative analgesics in children undergoing tonsillectomy.

The present study presents some limitations. A bigger sample size would allow for stronger and even more reliable results, and, to the same extent, it would be of interest to have a higher number of fathers partaking in the study. However, data was collected at the day of surgery, with this study possibly indicating that more mothers than father accompany their children to the Operation Room.

Nevertheless, to the best of our knowledge, this is the first study establishing an association between parental preoperative anxiety and adverse outcomes in children submitted to tonsillectomy, enhancing the importance of preoperative parental approaches and opening the door for further investigations on how these approaches might lower postoperative pain scores in children undergoing tonsillectomy.

## Conclusion

The present study establishes an association between preoperative parental anxiety, postoperative pain scores and the need for longer analgesic use in children undergoing tonsillectomy. This reinforces the importance of reducing parental anxiety and opens the door for further strategies to better post-tonsillectomy outcomes.

## Data Availability

Not applicable.

## Appendix I

### Instruções

Em baixo encontra uma série de frases que as pessoas costumam usar para se descreverem a si próprias. Leia cada uma delas e faça uma cruz (x) no número da direita que indique como se sente **agora**, isto é, **neste preciso momento**. Não há respostas certas nem erradas. Não leve muito tempo com cada frase, mas dê a resposta que melhor lhe parece descrever os seus sentimentos **neste momento.**

**1 – Nada 2 – Um pouco 3 – Moderadamente 4 – Muito**

1. Sinto-me calmo……………………………………………………………… 1 2 3 4

2. Sinto-me seguro……………………………………………………………. 1 2 3 4

3. Sinto-me tenso………………………………………………………………… 1 2 3 4

4. Sinto-me esgotado……………………………………………………………. 1 2 3 4

5. Sinto-me à vontade…………………………………………………………… 1 2 3 4

6. Sinto-me perturbado…………………………………………………………… 1 2 3 4

7. Presentemente, ando preocupado com desgraças que podem vir a acontecer… 1 2 3 4

8. Sinto-me satisfeito……………………………………………………………… 1 2 3 4

9. Sinto-me assustado…………………………………………………………….. 1 2 3 4

10. Estou descansado…………………………………………………………….. 1 2 3 4

11. Sinto-me confiante…………………………………………………………… 1 2 3 4

12. Sinto-me nervoso…………………………………………………………….. 1 2 3 4

13. Estou inquieto………………………………………………………………… 1 2 3 4

14. Sinto-me indeciso…………………………………………………………….. 1 2 3 4

15. Estou descontraído…………………………………………………………… 1 2 3 4

16. Sinto-me contente……………………………………………………………. 1 2 3 4

17. Estou preocupado…………………………………………………………….. 1 2 3 4

18. Sinto-me confuso…………………………………………………………….. 1 2 3 4

19. Sinto-me uma pessoa estável…………………………………………………. 1 2 3 4

20. Sinto-me bem………………………………………………………………… 1 2 3 4

### Instruções

Em baixo encontra uma série de frases que as pessoas costumam usar para se descreverem a si próprias. Leia cada uma delas e faça uma cruz (x) no número da direita que indique como se **sente em geral**. Não há respostas certas nem erradas. Não leve muito tempo com cada frase, mas dê a resposta que lhe parece descrever como se sente geralmente.

**1 – Quase nunca 2 – Algumas vezes 3 – Frequentemente 4 – Quase sempre**

21. Sinto-me bem………………………………………………………………… 1 2 3 4

22. Sinto-me nervoso e inquieto………………………………………………….. 1 2 3 4

23. Sinto-me satisfeito comigo próprio…………………………………………… 1 2 3 4

24. Quem me dera ser tão feliz como os outros parecem sê-lo…………………… 1 2 3 4

25. Sinto-me um falhado…………………………………………………………. 1 2 3 4

26. Sinto-me tranquilo……………………………………………………………. 1 2 3 4

27. Sou calmo, ponderado e senhor de mim mesmo……………………………… 1 2 3 4

28. Sinto que as dificuldades estão a acumular-se de tal forma que as não consigo resolver…………………………………………………………………………… 1 2 3 4

29. Preocupo-me demais com coisas que na realidade não têm importância……. 1 2 3 4

30. Sou feliz……………………………………………………………………… 1 2 3 4

31. Tenho pensamentos que me perturbam………………………………………. 1 2 3 4

32. Não tenho muita confiança em mim…………………………………………. 1 2 3 4

33. Sinto-me seguro……………………………………………………………… 1 2 3 4

34. Tomo decisões com facilidade……………………………………………….. 1 2 3 4

35. Muitas vezes sinto que não sou capaz………………………………………… 1 2 3 4

36. Estou contente………………………………………………………………… 1 2 3 4

37. Às vezes, passam-me pela cabeça pensamentos sem importância que me aborrecem …………………………………………………………………………………..…… 1 2 3 4

38. Tomo os desapontamentos tão a sério que não consigo afastá-los do pensamento ………………………………………………………………………………………………………………… 1 2 3 4

39. Sou uma pessoa estável………………………………………………………. 1 2 3 4

40. Fico tenso ou desorientado quando penso nas minhas preocupações e interesses mais recentes…………………………………………………………………………… 1 2 3 4
